# From Sprouting Angiogenesis to Erythrocytes Generation by Cancer Stem Cells: Evolving Concepts in Tumor Microcirculation

**DOI:** 10.1155/2014/986768

**Published:** 2014-08-04

**Authors:** Raafat S. Alameddine, Lana Hamieh, Ali Shamseddine

**Affiliations:** Division of Hematology and Oncology, Faculty of Medicine, American University of Beirut, P.O. Box 11-0236, Riad El Solh, Beirut 1107-2020, Lebanon

## Abstract

Angiogenesis is essential for tumor growth and metastasis. Over the last decades, a substantial progress has been achieved in defining different patterns of tumor microcirculation. Sprouting angiogenesis, the oldest model of microcirculation, is the de novo vessel formation from preexisting blood vessels. Vessel splitting and hijacking, also known, respectively, as intussusception and cooption, are alternative models that account for tumor resistance to antiangiogenic therapy. In addition to remodeling the microenvironment, the tumor cell can undergo intrinsic changes and survive hypoxic conditions by acquiring stem cell properties. In line with the concept of pluripotency, tumor cells can form vascular mimicry structures creating their own microcirculation despite a latent vessel growth. The recent identification of the polyploid giant cancer cells and tumor-derived erythrocytes is the most innovative survival mechanism in hypoxia and provides a potential target for more effective therapies.

## 1. Angiogenesis in Cancer

Angiogenesis is one of the oldest hallmarks of cancer. In the mid-twentieth century, the discovery of the highly vascular networks in solid tumors allowed a better understanding of tumor microcirculation. Over the last decades this fascinating feature has satisfied the pressing need for the oncology community in defining novel targets. More selective therapies were developed based on the better understood biologic features of the tumor. The class of antiangiogenic agents, the largest among targeted therapies, has been constantly growing as well as its approved indications (Table 1). In this review, we survey the earliest models of angiogenesis in the light of clinical experience with antiangiogenic agents. We explore the role of hypoxia in inducing an aggressive pattern in the tumor cell and acquisition of stem cell features. Finally we shed light on the recent findings of vascular mimicry structures and erythrocytes generation by pluripotent cancer stem cells.

## 2. Sprouting Angiogenesis: The Classical Model of Angiogenesis

In 1977, Folkman and Ausprunk proposed a model for sprouting of new vessels from preexisting vasculature. In sprouting angiogenesis, endothelial cells redefine their role and assume a new configuration to maintain the perfusion of the growing tumor. Factors released by tumor cells, such as vascular endothelial growth factor (VEGF), follicular growth factor (FGF), platelet derived growth factor (PDGF), stimulate a cascade of endothelial changes leading to de novo vessel formation [1]. The whole process starts by the disintegration of the basement membrane followed by the loosening of the intercellular junctions that link endothelial cells [2]. Once free, the endothelial cells rearrange and polarize to invade the surrounding extracellular matrix and finally seal gaps and produce a basement membrane lining the growing vessel. Angiogenesis is regulated by a wide array of receptors and ligands participating all together in an organized crosstalk [1, 3]. The family of VEGF and its receptors VEGFRs has been recognized as the power horse driving de novo vessel formation [1]. VEGF-A and its receptor VEGFR-2 are of particular importance [4–7]. The activation of the VEGFR is translated by the dimerization of the tyrosine kinase domains and the triggering of intracellular signaling cascades ending by endothelial proliferation and budding of new vessels [8].

## 3. Alternative Models of Endothelium-Dependent Microcirculation

Clinical experience with VEGF targeting therapies unveiled the caveats in the conventional model of angiogenesis in cancer. A telltale story is the short-term improvement in the progression free survival with the use of bevacizumab, a monoclonal antibody blocking VEGF-A, in breast cancer followed by rebound aggressiveness and metastasis shortening the overall survival [9, 10]. Poor clinical response with antiangiogenic therapy invited a reconsideration of alternative models of tumor microcirculation. In mice inoculated with cancer cells, short-term treatment with a multireceptor tryosine kinase inhibitor resulted in metastatic conditioning with accelerated metastasis and death of the mice [11]. In glioblastoma and pancreatic neuroendocrine tumor models, Pàez-Ribes et al. observed that pharmacological or genetic silencing of VEGF-A surprisingly translated into a higher invasion and distant metastasis [12]. Interestingly enough, the relapsing tumor was hypoxic and devoid of vasculature. This association between hypoxia and a more aggressive behavior corroborates a series of clinical and experimental studies [13–16]. Hypoxia involves a cascade of molecular and phenotypic changes in the tumor cell enhancing its ability to grow, invade, and metastasize. These changes can evoke endothelium dependent models such as vessel cooption and intussusception.

### 3.1. Vessel Cooption

Vessel cooption is one the earliest mechanisms giving tumor cells access to nutrients and oxygen from the intravascular space. Driven by low oxygen tension, tumor cells abut the walls of existing vessels as preliminary step before forming a new full-blown vasculature [17]. Densely vascularized organs such as lung, liver, and brain provide an excellent niche for tumor cells to hijack preexisting vessels and form solitary metastases [18, 19]. Vessel cooption is one of the mechanisms of acquired resistance to antiangiogenic therapy. Treatment of brain metastases with ZD6474, an antiangiogenic agent, was shown to induce a marked rise in vessel cooption [20]. In patients with renal cell carcinoma, treatment with Sunitinib was associated with extensive necrosis at the tumor center in contrast to the rim highly rich with viable tumor cells [21, 22]. Deregulation in cellular energetic is essential feature of the coopting tumor cells [23]. In vessel cooption, tumor cells highly depend on an active mitochondrial metabolism, protein synthesis, and cell cycle. On the opposite side, angiogenic tumors are highly dependent on remodeling of the microenvironment as reflected by the elevated expression of membrane vesicles, integrins, and growth factors [23]. Vessel cooption can also account for the differential response to antiangiogenic therapy among different tumors. Liver metastases from breast cancer origin have been shown to depend more on cooption of the liver vasculature than those from colorectal origin [24]. The latter finding can account for the higher efficacy of VEGF targeting therapy in metastatic colorectal cancer compared to metastatic breast cancer.

### 3.2. Intussusception

Intussusception is another adaptive response of the tumor cells to stress and hypoxia. First observed in neonatal rat lungs in 1986, and in colon cancer in 1996, intussusceptive vessel growth results from change in shear stress and remodeling of the vascular network [25, 26]. Switch from sprouting angiogenesis to intussusceptive angiogenesis is an escape strategy from radiation induced damage to the tumor vasculature [27]. Despite exposure to antiangiogenic therapy and subsequent significant decrease in microvascular density, mammary carcinoma had a rapid recovery post treatment cessation, in virtue of intussusceptive pruning. Intussusceptive vessel growth involves bilateral centripetal protrusion of opposite endothelial cells lining the vessel wall [28]. Once in contact, the opposite vessel walls fuse and tiny apertures in endothelial lining of the vessel walls form, ultimately leading to the splitting of the two newly formed vessels [28, 29]. In contrast to sprouting angiogenesis, splitting angiogenesis is an energy conserving mechanism as it does not dependent on a high rate of proliferation or on basement membrane degradation and invasion, therefore saving energy and permitting survival of the tumor despite hypoxia and stress [25, 28, 30, 31].

## 4. Epithelial Mesenchymal Transition and Underlying Molecular Pathways

Besides the remodeling in vascular network, the tumor cell can undergo radical morphological and functional changes to survive hypoxia and stress. The epithelial mesenchymal transition (EMT) is a biological model that accounts for tumor cell adaptation to hypoxia in an endothelium-independent manner [32]. EMT involves a cascade of changes at different levels including rearrangement of markers from cell surface to nucleus, cytoskeleton remodeling, and acquisition of mesenchymal identity [33]. The cell gradually loses the epithelial tags. [34]. The intracellular scaffold undergoes also a radical transformation as its constituents change from actin microfilaments to vimentin and its polarity from well-defined apicobasal to a new flexible geometry more commensurate with the new invasive identity. Also, the production of matrix metalloproteinases (MMPs) is increased, and the extracellular matrix (ECM) production line shifts from the basement membrane towards interstitial filaments [35, 36]. EMT changes ultimately lead to the cell acquisition of stem cell properties conferring survival advantage to the transforming cell. EMT depends on a range of molecular pathways involved in early morphogenesis and accounts for resistance to chemotherapy including NF-*κ*B, PI3 K/Akt/mTOR, Notch, Wnt/*β*-catenin, and Hedgehog signaling [33].

### 4.1. Nuclear Factor Kappa B (NF-*κ*B)

The NF-*κ*B pathway is one of the pathways most intimately linked to the tumorigenesis and survival in hypoxia [37]. The NF-*κ*B is located the cytoplasm as an inactive heterodimeric protein that gets activated and releases its subunits to the nuclear compartments where they upregulate the expression of many oncogenic proteins [38]. Hypoxia promotes the phosphorylation and degradation of the inhibitor of kappa B (I*κ*Ba), thus leading to activation of NF-*κ*B and subsequent transition to a mesenchymal phenotype [39, 40]. In pancreatic cancer cell lines, hypoxia was shown to induce the acquisition of mesenchymal properties by epithelial cells through the production of HIF-alpha [41]. Upon inhibition of NF-*κ*B, a regression was observed in the aggression induced by hypoxia exposure. Even under normoxic conditions, the same pattern was reproduced indicating a critical role for NF-*κ*B in translating of HIF overexpression into phenotypical changes [41].

### 4.2. The PI3K mTOR Pathway

The PI3K/Akt/mTOR axis also regulates hypoxia induced EMT. Blockade of PI3K pathway in hepatocellular carcinoma (HCC) abrogated all the EMT changes induced by hypoxia [42]. The same pattern was reproduced in prostate and ovarian cancer cell lines where the addition of PI3K inhibitor abolished the HIF-1 *α* activation of Smad2/3 and Akt/GSK-3*β*, decreased the expression of Snail of transcription and translation, and prevented the decrease in E-cadherin expression and increased cell motility [43].

### 4.3. The Notch Family of Ligands

Notch signaling is implicated in the cellular response to hypoxia. The Notch family is composed of four receptors and five membrane ligands. Interplay among all of these molecules results in intercellular communication through direct cell-cell contact [44]. Notch signaling is triggered by the binding of Notch ligand to the Notch receptor in the counterpart cell. In oral squamous cell carcinoma, Ishida, Hijioka noted the upregulation of Notch receptors, ligands, and target genes in hypoxia, the fact that translated in increased invasion and cellular motility. The expression of the epithelial marker E-cadherin, a key event in EMT, was decreased in hypoxia. Addition of Notch-specific inhibitor abrogated all the hypoxia induced cellular changes [45, 46]. In breast cancer cell lines, blockade of Notch pathway repressed the expression of Snail and Slug and decreased acquisition of mesenchymal features under hypoxia conditions [46].

### 4.4. The Wnt/*β*-Catenin Pathway

The Wnt/*β*-catenin signalling system is one of the main mediators of hypoxia induced EMT changes [47]. The Wnt/*β*-catenin is a highly conserved signaling pathway and operates through three different pathways: the canonical pathway involved in the regulation of gene transcription and the noncanonical pathways involved in regulation of intracellular calcium and cytoskeleton [48, 49]. Overexpression of HIF-alpha in prostate cancer cell lines correlated with increased beta catenin protein expression and resulted in enhanced typical EMT changes [50]. In colorectal cancer, hypoxia activated *β*-catenin and Nur77 with a subsequent increase in cell invasion and migration [51]. In HCC, beta catenin expression was shown to be a key mediator of hypoxia induced EMT, the latter correlated in both in vitro and in vivo models [52]. On microarray analysis of HCC samples, beta catenin and HIF alpha were coexpressed and significantly associated with a shorter survival.

### 4.5. The Hedgehog Signaling Pathway

The Hedgehog (Hh) pathway also promotes EMT in response to hypoxia. Three ligands compose the family of Hh signals: the Sonic Hedgehog (Shh), the Indian Hedgehog (Ihh), and the Desert Hedgehog (Dhh) [53]. Pretreatment of gastric cancer cells with Shh upregulates EMT, decreases E-cadherin, and induces tumor invasiveness in vitro [54]. Analysis of gastric cancer tissues revealed significant correlation between Shh expression, EMT, and lymphangiogenesis [54]. Hypoxia effect on cell invasion was negated by antagonism of Smoothened (SMO), the cell surface receptor in Hh pathway. Lei et al. investigated the effect of inhibition of the noncanonical Hh axis on hypoxia induced EMT. The authors found that hypoxia effect on GLI1, a key transcription factor in the Hh pathway, is not restricted to SMO and involves other factors [55]. In a separate experiment on pancreatic ductal adenocarcinoma, Onishi et al. found that increase in cell invasiveness is affected by hypoxia stimulation of Hh axis in a process involving SMO, Gli1 [56].

## 5. Vascular Mimicry: Insight into Tumor Plasticity

In 1999, the landmark work of Maniotis et al. paved the way for a novel understanding of an endothelium-independent tumor microcirculation [57]. Uveal melanoma cells were found to align in the form of channels gaining access to the systemic circulation by expressing the embryonic genetic repertoire. In contrast to the classical erratic growth of vessels in sprouting angiogenesis, these vascular structures are part of a highly organized network of vessels devoid of any endothelial lining [57]. Vasculogenic mimicry involves several signaling pathways contributing to embryonic vasculogenesis and reflecting an acquired pluripotency in melanoma cells [58]. Additionally, it highly depends on the elevated expression of VEGFR-2. Francescone et al. examined the knock-down of VEGFR-2 or the selective blockade of this receptor in two glioblastoma cell lines U87 and GSDC and reported the detrimental effects of these manipulations on vascular formation [59]. The intracellular cascades mediating proliferation and migration were also significantly affected. The same results were not replicated when using the monoclonal antibody to VEGF-A Indicating that VEGF-A independent mechanisms account for vascular formation in the glioblastoma models [59, 60]. Moreover in another study on glioblastoma cell lines done by Scully et al., vascular channels were found to selectively express platelet derived growth factor receptor (PDGFR) *β*, smooth muscle markers, and VEGFR-2 with a striking lack of expression of CD31 and VE-cadherin [60]. Another feature of vascular mimicry structures is the unique crosstalk exhibited with the microenvironment through upregulation of proteases such as matrix metalloproteinases (MMPs) 1, 2, 9 and MT1-MMP (MMP-14) or basement membrane proteins such as laminin 5 (Ln-5, gamma 2 chain) [61]. Additionally, the activation of antiapoptotic proteins was found to be essential for generation of VM structures in response to hypoxia. Indeed, Bcl-2 silencing by means of si-RNA depressed the VM response to hypoxia [62]. Central to the vascular mimicry formation as well is the interaction between the cell surface marker VE-cadherin and its receptor the Ephrin type-A receptor 2. The former is known as CD144 is an adhesion molecule playing a vital role for maintaining interendothelial cell contact. The latter is a transmembrane protein kinase closely related to the MAP/ERK kinase cascade. The colocalization between these two molecules on the cell surface is crucial for conductance of proper intracellular signaling in the endothelium mimicking tumor cell with all the ensuing alteration in protein synthesis necessary for fulfillment of this new task [63]. Ewing sarcoma cells also formed VM structures allowing perfusion of tumor cells. And hypoxia is essential in giving these tumor cells plasticity. Given the high prevalence of vascular mimicry structures in Ewing sarcoma, this disease is a prototype for studying of formation of such unique tumor based conduits and defining potential targets for therapy [64]. In prostate cancer, high grade tumors exhibit abundance of vascular mimicry structures, and these cells might be located even in the close vicinity of conventional endothelium-lined blood vessels [65]. In gliomas, the presence of vascular mimicry correlated with a worse prognosis. In fact, vascular mimicry structures allowed a better tumor perfusion even in areas with lower vessel microdensity [66]. In colon cancer, the presence of vasculogenic mimicry was associated with poor anatomical risk factors and a shorter survival [67].

## 6. Polyploid Giant Cancer Cells (PGCCs) and Erythrocytes Generation

An effective tumor microcirculation rests on a sustainable access to the systemic circulation and a continuous supply with red blood cells to meet the tumor needs. In contrast to tumor angiogenesis, little is known about tumor erythropoiesis. Until recently, the bone marrow has been considered the sole source for erythrocytes circulating in the tumor microenvironment. However, the immunohistochemical detection of fetal forms of hemoglobin in various solid tumors suggested the presence of an alternative origin for high affinity hemoglobin [68]. The tumor stroma was proposed to a potential source of erythrocytes [69]. The recent finding of erythrocytes generation by cancer cells under conditions of hypoxia stirred a lot of interest. In the absence of angiogenesis, cancer cells can produce erythrocytes with embryonic and fetal forms of hemoglobin that carry oxygen with high affinity [70]. The work of Zhang et al. has substantially contributed to the delineation of the steps underlying formation of erythrocytes from cancer cells [70]. Zhang et al. reported the presence of polyploid giant cancer cells (PGCCs) in cells exposed to hypoxia and stress conditions. In virtue of their pluripotency, these cells represent a salvage pathway to survive genotoxic insults [71].

PGCCs are a special population of human tumor cells characterized by a large cytoplasm, aneuploidy, and multiple nuclei [72]. PGCCs are survivors from mitotic catastrophe, whereby failure of induction of programmed cell death results in the formation of aneuploid cells [73]. In states of hypoxia and stress, cancer cells fuse by means of endoreduplication typical of lower eukaryotes and form giant cells with multiple copies of the genome [74].

The presence of aneuploid cells and PGCCs is associated with an increased risk for malignant transformation [73]. In different malignancies, PGCCs are a marker of increased stem cell properties, EMT, and a more aggressive biology [75–78].

The novel feature of erythrocytes production by PGCCs was validated in different experiments. Treatment of breast cancer cells with CoCl2, a hypoxia-mimicking agent, resulted in the selective survival of giant cancer cells similar in size to PGCCs [70]. After multiple rounds of exposure to hypoxia, PGCCs gave rise to spheroids expressing cancer stem cell markers. On histological examination, the newly formed spheroids shared with erythrocytes morphological features in terms of size, biconcavity, and absence of nuclei. Spheroids were positively stained for fetal and embryonic forms of hemoglobin, all consistent with a highly efficient oxygen delivery system to the growing tumor. Similarly ovarian fibroblasts cancer cell lines exposed to hypoxia produced erythrocytes with a predominance of the embryonic and fetal forms of hemoglobin. In a separate experiment, breast cancer cell lines treated with a microtubule stabilizer “paclitaxel” went into a mitotic arrest [71]. Subsequently, they formed PGCCs that used peculiar modes of replication to develop into a variety of stromal cells including erythroid cells, all conferring resistance to treatment with paclitaxel [71]. In human glioma PGCCs identified by morphological features in the tumor tissue bank were inoculated into chicken embryonating eggs. Immunohistochemical staining was performed for *β*/*γ*/*ε*/*δ* chain on red cells bodies inside PGCCs, and red cell bodies turned out to be erythrocytes [76]. All this evidence suggests that cancer cells can be endowed with marvelous features such as erythrocytes generation when challenged with hypoxia.

## 7. Future Directions

**Figure 1:**
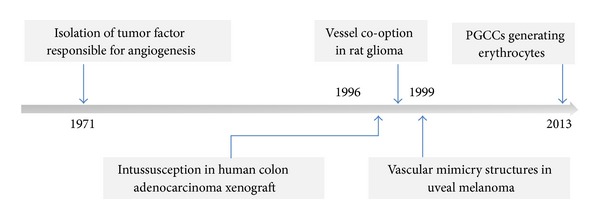
Historical overview of evolution of models of angiogenesis.

The ability of cancer cells to form new blood vessels was a groundbreaking finding in the early seventies (Figure 1). The alternative models of angiogenesis provided explanation for the failure of antiangiogenic therapy. Subsequently, the demonstration of VM structures was another major leap in understanding the growth and survival of tumor cells in the absence of neo-vessel formation. It illustrates the tumor cell plasticity through the induction of molecular pathways involved in embryogenesis and early morphogenesis, also found in cancer stem cell formation. The most recent finding of tumor microcirculation is the erythrocytes generation by PGCCs. Erythrocytes generation by cancer stem cells will invite a lot of future research to better understand the molecular pathways underlying the genesis of tumor-derived erythrocytes and to potentially develop novel therapies targeting them.

## Figures and Tables

**Table 1:** List of FDA approved antiangiogenic agents.

Antiangiogenic agent	Pharmacological class	FDA approvals
Bevacizumab	Monoclonal antibody	(i) Metastatic colorectal cancer (ii) Metastatic non-small nonsquamous cell lung carcinoma (iii) Renal cell carcinoma (in combination with interferon-alpha immunotherapy) (iv) Glioblastoma

Pazopanib	Multireceptor tyrosine kinase inhibitor	(i) Renal cell carcinoma (ii) Soft tissue sarcoma

Sunitinib	Multireceptor tyrosine kinase inhibitor	(i) Second line in gastrointestinal stromal tumor after imatinib exposure (ii) Advanced renal cell carcinoma (iii) Progressive pancreatic neuroendocrine tumors

Vandetanib	Multireceptor tyrosine kinase inhibitor	Medullary thyroid carcinoma

Sorafenib	Multireceptor tyrosine kinase inhibitor	(i) Renal cell carcinoma (ii) Hepatocellular carcinoma

Ziv-aflibercept	Monoclonal antibody	Second line in metastatic colorectal cancer

Ramucirumab	Monoclonal antibody	Metastatic esophageal, gastric, or gastroesophageal junction carcinoma

## References

[1] Otrock ZK, Mahfouz RAR, Makarem JA, Shamseddine AI (2007). Understanding the biology of angiogenesis: review of the most important molecular mechanisms. *Blood Cells, Molecules, and Diseases*.

[2] Carmeliet P (2000). Mechanisms of angiogenesis and arteriogenesis. *Nature Medicine*.

[3] Alameddine RS, Otrock ZK, Awada A, Shamseddine A (2013). Crosstalk between HER2 signaling and angiogenesis in breast cancer: molecular basis, clinical applications and challenges. *Current Opinion in Oncology*.

[4] Jakeman LB, Armanini M, Phillips HS, Ferrara N (1993). Developmental expression of binding sites and messenger ribonucleic acid for vascular endothelial growth factor suggests a role for this protein in vasculogenesis and angiogenesis. *Endocrinology*.

[5] Ferrara N, Gerber H, LeCouter J (2003). The biology of VEGF and its receptors. *Nature Medicine*.

[6] Cross MJ, Dixelius J, Matsumoto T, Claesson-Welsh L (2003). VEGF-receptor signal transduction. *Trends in Biochemical Sciences*.

[7] Neufeld G, Tessler S, Gitay-Goren H, Cohen T, Levi BZ (1994). Vascular endothelial growth factor and its receptors. *Progress in Growth Factor Research*.

[8] Hood JD, Meininger CJ, Ziche M, Granger HJ (1998). VEGF upregulates ecNOS message, protein, and NO production in human endothelial cells. *American Journal of Physiology—Heart and Circulatory Physiology*.

[9] Miller K, Wang M, Gralow J (2007). Paclitaxel plus bevacizumab versus paclitaxel alone for metastatic breast cancer. *The New England Journal of Medicine*.

[10] Kerbel RS (2008). Tumor angiogenesis. *The New England Journal of Medicine*.

[11] Ebos JML, Lee CR, Cruz-Munoz W, Bjarnason GA, Christensen JG, Kerbel RS (2009). Accelerated metastasis after short-term treatment with a potent inhibitor of tumor angiogenesis. *Cancer Cell*.

[12] Pàez-Ribes M, Allen E, Hudock J (2009). Antiangiogenic therapy elicits malignant progression of tumors to increased local invasion and distant metastasis. *Cancer Cell*.

[13] Brizel DM, Sibley GS, Prosnitz LR, Scher RL, Dewhirst MW (1997). Tumor hypoxia adversely affects the prognosis of carcinoma of the head and neck. *International Journal of Radiation Oncology Biology Physics*.

[14] Höckel M, Schlenger K, Aral B, Mitze M, Schäffer U, Vaupel P (1996). Association between tumor hypoxia and malignant progression in advanced cancer of the uterine cervix. *Cancer Research*.

[15] Walenta S, Salameh A, Lyng H (1997). Correlation of high lactate levels in head and neck tumors with incidence of metastasis. *The American Journal of Pathology*.

[16] Pitson G, Fyles A, Milosevic M, Wylie J, Pintilie M, Hill R (2001). Tumor size and oxygenation are independent predictors of nodal diseases in patients with cervix cancer. *International Journal of Radiation Oncology Biology Physics*.

[17] Holash J, Maisonpierre PC, Compton D (1999). Vessel cooption, regression, and growth in tumors mediated by angiopoietins and VEGF. *Science*.

[18] Donnem T, Hu J, Ferguson M (2013). Vessel co-option in primary human tumors and metastases: an obstacle to effective anti-angiogenic treatment?. *Cancer Medicine*.

[19] Vermeulen PB, Colpaert C, Salgado R (2001). Liver metastases from colorectal adenocarcinomas grow in three patterns with different angiogenesis and desmoplasia. *Journal of Pathology*.

[20] Leenders WPJ, Küsters B, Verrijp K (2004). Antiangiogenic therapy of cerebral melanoma metastases results in sustained tumor progression via vessel co-option. *Clinical Cancer Research*.

[21] Smith AD, Shah SN, Rini BI, Lieber ML, Remer EM (2010). Morphology, Attenuation, Size, and Structure (MASS) criteria: assessing response and predicting clinical outcome in metastatic renal cell carcinoma on antiangiogenic targeted therapy. *American Journal of Roentgenology*.

[22] Welti JC, Powles T, Foo S (2012). Contrasting effects of sunitinib within in vivo models of metastasis. *Angiogenesis*.

[23] Hu J, Bianchi F, Ferguson M (2005). Gene expression signature for angiogenic and nonangiogenic non-small-cell lung cancer. *Oncogene*.

[24] Stessels F, van den Eynden G, van der Auwera I (2004). Breast adenocarcinoma liver metastases, in contrast to colorectal cancer liver metastases, display a non-angiogenic growth pattern that preserves the stroma and lacks hypoxia. *British Journal of Cancer*.

[25] Caduff JH, Fischer LC, Burri PH (1986). Scanning electron microscope study of the developing microvasculature in the postnatal rat lung. *Anatomical Record*.

[26] Patan S, Munn LL, Jain RK (1996). Intussusceptive microvascular growth in a human colon adenocarcinoma xenograft: a novel mechanism of tumor angiogenesis. *Microvascular Research*.

[27] Hlushchuk R, Riesterer O, Baum O (2008). Tumor recovery by angiogenic switch from sprouting to intussusceptive angiogenesis after treatment with PTK787/ZK222584 or ionizing radiation. *American Journal of Pathology*.

[28] Djonov V, Baum O, Burri PH (2003). Vascular remodeling by intussusceptive angiogenesis. *Cell and Tissue Research*.

[29] Djonov V, Schmid M, Tschanz SA, Burri PH (2000). Intussusceptive angiogenesis: its role in embryonic vascular network formation. *Circulation Research*.

[30] Collen A, Hanemaaijer R, Lupu F (2003). Membrane-type matrix metalloproteinase-mediated angiogenesis in a fibrin-collagen matrix. *Blood*.

[31] Prager GW, Breuss JM, Steurer S, Mihaly J, Binder BR (2004). Vascular endothelial growth factor (VEGF) induces rapid prourokinase (pro-uPA) activation on the surface of endothelial cells. *Blood*.

[32] Brahimi-Horn MC, Chiche J, Pouysségur J (2007). Hypoxia and cancer. *Journal of Molecular Medicine*.

[33] Bao B, Azmi AS, Ali S (2012). The biological kinship of hypoxia with CSC and EMT and their relationship with deregulated expression of miRNAs and tumor aggressiveness. *Biochimica et Biophysica Acta*.

[34] Asiedu MK, Ingle JN, Behrens MD, Radisky DC, Knutson KL (2011). TGF*β*/TNF*α*-mediated epithelial-mesenchymal transition generates breast cancer stem cells with a claudin-low phenotype. *Cancer Research*.

[35] Hay ED (1995). An overview of epithelio-mesenchymal transformation. *Acta Anatomica*.

[36] Hugo H, Ackland ML, Blick T (2007). Epithelial—mesenchymal and mesenchymal—epithelial transitions in carcinoma progression. *Journal of Cellular Physiology*.

[37] Shannon AM, Bouchier-Hayes DJ, Condron CM, Toomey D (2003). Tumour hypoxia, chemotherapeutic resistance and hypoxia-related therapies. *Cancer Treatment Reviews*.

[38] Schreck R, Albermann K, Baeuerle PA (1992). Nuclear factor *κβ*: an oxidative stress-responsive transcription factor of eukaryotic cells (a review). *Free Radical Research Communications*.

[39] Koong AC, Chen EY, Giaccia AJ (1994). Hypoxia causes the activation of nuclear factor *κ*B through the phosphorylation of I*κ*B*α* on tyrosine residues. *Cancer Research*.

[40] Huber MA, Azoitei N, Baumann B (2004). NF-*κ*B is essential for epithelial-mesenchymal transition and metastasis in a model of breast cancer progression. *Journal of Clinical Investigation*.

[41] Cheng Z, Sun B, Wang S (2011). Nuclear factor-*κ*B-dependent epithelial to mesenchymal transition induced by HIF-1*α* activation in pancreatic cancer cells under hypoxic conditions. *PLoS ONE*.

[42] Yan W, Fu Y, Tian D (2009). PI3 kinase/Akt signaling mediates epithelial-mesenchymal transition in hypoxic hepatocellular carcinoma cells. *Biochemical and Biophysical Research Communications*.

[43] Lin G, Gai R, Chen Z (2014). The dual PI3K/mTOR inhibitor NVP-BEZ235 prevents epithelial-mesenchymal transition induced by hypoxia and TGF-beta1. *European Journal of Pharmacology*.

[44] Miele L (2006). Notch signaling. *Clinical Cancer Research*.

[45] Ishida T, Hijioka H, Kume K, Miyawaki A, Nakamura N (2013). Notch signaling induces EMT in OSCC cell lines in a hypoxic environment. *Oncology Letters*.

[46] Chen J, Imanaka N, Griffin JD (2010). Hypoxia potentiates Notch signaling in breast cancer leading to decreased E-cadherin expression and increased cell migration and invasion. *British Journal of Cancer*.

[47] Fodde R, Brabletz T (2007). Wnt/*β*-catenin signaling in cancer stemness and malignant behavior. *Current Opinion in Cell Biology*.

[48] Nusse R, Varmus HE (1992). Wnt genes. *Cell*.

[49] Logan CY, Nusse R (2004). The Wnt signaling pathway in development and disease. *Annual Review of Cell and Developmental Biology*.

[50] Jiang YG, Luo Y, He DL (2007). Role of Wnt/*β*-catenin signaling pathway in epithelial-mesenchymal transition of human prostate cancer induced by hypoxia-inducible factor-1*α*. *International Journal of Urology*.

[51] To SK, Zeng WJ, Zeng JZ, Wong AS (2014). Hypoxia triggers a Nur77-beta-catenin feed-forward loop to promote the invasive growth of colon cancer cells. *The British Journal of Cancer*.

[52] Liu L, Zhu X, Wang W (2010). Activation of *β*-catenin by hypoxia in hepatocellular carcinoma contributes to enhanced metastatic potential and poor prognosis. *Clinical Cancer Research*.

[53] Di Magliano MP, Hebrok M (2003). Hedgehog signalling in cancer formation and maintenance. *Nature Reviews Cancer*.

[54] Yoo YA, Kang MH, Lee HJ (2011). Sonic hedgehog pathway promotes metastasis and lymphangiogenesis via activation of Akt, EMT, and MMP-9 pathway in gastric cancer. *Cancer Research*.

[55] Lei J, Ma J, Ma Q (2013). Hedgehog signaling regulates hypoxia induced epithelial to mesenchymal transition and invasion in pancreatic cancer cells via a ligand-independent manner. *Molecular Cancer*.

[56] Onishi H, Kai M, Odate S (2011). Hypoxia activates the hedgehog signaling pathway in a ligand-independent manner by upregulation of Smo transcription in pancreatic cancer. *Cancer Science*.

[57] Maniotis AJ, Folberg R, Hess A (1999). Vascular channel formation by human melanoma cells *in vivo* and *in vitro*: vasculogenic mimicry. *The American Journal of Pathology*.

[58] Hendrix MJC, Seftor EA, Hess AR, Seftor REB (2003). Vasculogenic mimicry and tumour-cell plasticity: lessons from melanoma. *Nature Reviews Cancer*.

[59] Francescone R, Scully S, Bentley B (2012). Glioblastoma-derived tumor cells induce vasculogenic mimicry through Flk-1 protein activation. *The Journal of Biological Chemistry*.

[60] Scully S, Francescone R, Faibish M (2012). Transdifferentiation of glioblastoma stem-like cells into mural cells drives vasculogenic mimicry in glioblastomas. *Journal of Neuroscience*.

[61] Seftor REB, Seftor EA, Koshikawa N (2001). Cooperative interactions of laminin 5 *γ*2 chain, matrix metalloproteinase-2, and membrane type-1-matrix/metalloproteinase are required for mimicry of embryonic vasculogenesis by aggressive melanoma. *Cancer Research*.

[62] Zhao N, Sun BC, Sun T (2012). Hypoxia-induced vasculogenic mimicry formation via VE-cadherin regulation by Bcl-2. *Medical Oncology*.

[63] Hess AR, Seftor EA, Gruman LM, Kinch MS, Seftor REB, Hendrix MJC (2006). VE-cadherin regulates EphA2 in aggressive melanoma cells through a novel signaling pathway: implications for vasculogenic mimicry. *Cancer Biology and Therapy*.

[64] van der Schaft DWJ, Hillen F, Pauwels P (2005). Tumor cell plasticity in Ewing sarcoma, an alternative circulatory system stimulated by hypoxia. *Cancer Research*.

[65] Sharma N, Seftor REB, Seftor EA (2002). Prostatic tumor cell plasticity involves cooperative interactions of distinct phenotypic subpopulations: role in vasculogenic mimicry. *Prostate*.

[66] Chen YS, Chen ZP (2014). Vasculogenic mimicry: a novel target for glioma therapy. *Chinese Journal of Cancer*.

[67] Baeten CIM, Hillen F, Pauwels P, de Bruine AP, Baeten CGMI (2009). Prognostic role of vasculogenic mimicry in colorectal cancer. *Diseases of the Colon and Rectum*.

[68] Wolk M (2014). Considerations on the possible origins of fetal hemoglobin cells produced in developing tumors. *Stem Cells and Development*.

[69] Szabo E, Rampalli S, Risueño RM (2010). Direct conversion of human fibroblasts to multilineage blood progenitors. *Nature*.

[70] Zhang S, Mercado-Uribe I, Liu J (2013). Generation of erythroid cells from fibroblasts and cancer cells in vitro and in vivo. *Cancer Letters*.

[71] Zhang S, Mercado-Uribe I, Liu J (2014). Tumor stroma and differentiated cancer cells can be originated directly from polyploid giant cancer cells induced by paclitaxel. *International Journal of Cancer*.

[72] Yang T, Rycaj K, Liu Z, Tang DG (2014). Cancer stem cells: constantly evolving and functionally heterogeneous therapeutic targets. *Cancer Research*.

[73] Castedo M, Perfettini J, Roumier T, Andreau K, Medema R, Kroemer G (2004). Cell death by mitotic catastrophe: a molecular definition. *Oncogene*.

[74] Zhang D, Wang Y, Zhang S (2014). Asymmetric cell division in polyploid giant cancer cells and low eukaryotic cells. *BioMed Research International*.

[75] Erenpreisa J, Cragg MS (2013). Three steps to the immortality of cancer cells: senescence, polyploidy and self-renewal. *Cancer Cell International*.

[76] Qu Y, Zhang L, Rong Z, He T, Zhang S (2013). Number of glioma polyploid giant cancer cells (PGCCs) associated with vasculogenic mimicry formation and tumor grade in human glioma. *Journal of Experimental & Clinical Cancer Research*.

[77] Illidge TM, Cragg MS, Fringes B, Olive P, Erenpreisa JA (2000). Polyploid giant cells provide a survival mechanism for p53 mutant cells after DNA damage. *Cell Biology International*.

[78] Zhang S, Mercado-Uribe I, Xing Z, Sun B, Kuang J, Liu J (2014). Generation of cancer stem-like cells through the formation of polyploid giant cancer cells. *Oncogene*.

